# Effects of the Interaction between Affective Temperaments and BIS/BAS on Depressive Symptoms in Individuals with Major Depressive Disorder

**DOI:** 10.3390/ijerph192315841

**Published:** 2022-11-28

**Authors:** Kuniyoshi Toyoshima, Jiro Masuya, Miki Ono, Mina Honyashiki, Shogo Hashimoto, Ichiro Kusumi, Takeshi Inoue

**Affiliations:** 1 Department of Psychiatry, Graduate School of Medicine, Hokkaido University, Sapporo 060-8638, Japan; 2 Department of Psychiatry, Tokyo Medical University, Tokyo 160-0023, Japan

**Keywords:** affective temperaments, behavioral activation system, behavioral inhibition system, depression, interaction, major depressive disorder

## Abstract

Depressive symptoms (DepS) associated with major depressive disorder (MDD) are influenced by affective temperaments (ATs), behavioral inhibition system (BIS), and behavioral activation system (BAS). However, the effect of interactions between ATs and BIS/BAS on DepS in MDD remains poorly understood. Herein, we aimed to investigate the effects of these interactions. The Temperament Evaluation of Memphis, Pisa, Paris, and San Diego Auto-questionnaire (TEMPS-A), BIS/BAS questionnaire, and Patient Health Questionnaire-9 (PHQ-9) were used to evaluate ATs, BIS/BAS, and DepS, respectively, in 90 participants with MDD. Data were analyzed using hierarchical multiple regression analysis to assess the interaction effect. The interaction (*β* = 0.199, *p* < 0.05) between depressive temperament (DepT) (*β* = 0.319, *p* < 0.01) and BIS scores (*β* = 0.300, *p* < 0.01) exhibited a significant positive effect on DepS (Δ*R*^2^ = 0.038, *p* < 0.05). However, the interaction between ATs and BAS scores did not exhibit a significant effect on DepS. Our findings suggest that interactions between BIS sensitivity and DepT worsen DepS in individuals with MDD. Hence, to manage DepS associated with BIS sensitivity and DepT, evaluating their interaction may be useful in daily clinical practice. This study presents important insights into MDD psychopathology.

## 1. Introduction

Major depressive disorder (MDD) is a common and multifactorial illness, with a heritability of 30–50% [1,2,3]. The onset of MDD negatively impacts the quality of life and psychosocial functioning of individuals. Furthermore, the burden of MDD is high [4]. The following correlated risk factors have been reported in MDD: parental depression, being female, neuroticism, childhood maltreatment, dysfunctional cognitions, interpersonal dysfunction, stressful life events, insomnia, and chronic or severe physical illness [5,6,7,8,9,10]. Furthermore, vulnerability and resilience are associated with MDD onset [11,12]. However, the interrelations of the abovementioned correlated risk factors remain unclear. Notably, the association among temperament, personality, and depression onset in terms of the psychopathology of MDD is yet to be clarified.

Affective temperaments (ATs) are trait markers that demonstrate potential mood psychopathology and greatly impact people with mood disorders throughout their lives [13,14]. The domains of ATs, such as depressive (DepT), cyclothymic (CycT), hyperthymic (HypT), irritable (IrrT), and anxious (AnxT) temperaments, are identified using the Temperament Evaluation Memphis, Pisa, Paris, and San Diego Auto-questionnaire (TEMPS-A) [15,16]. Rovai et al. [17] reported a positional gap in clinical psychiatry between HypT, which is the most functional temperament, and CycT, DepT, IrrT, and AnxT, which have been more closely correlated with mood disorders. Although cultural factors can affect the expression of ATs [14], CycT and IrrT develop on a continuum of temperaments, from healthy through MDD to bipolar disorder [18]. CycT and AnxT are factors of differential diagnosis between MDD and bipolar disorder in Japan and are associated with the diagnosis of bipolar disorder [19]. In Japanese patients with MDD, the severity of depressive symptoms (DepS) is considered directly influenced by DepT, CycT, IrrT, and AnxT [20]. A previous study in Japan suggests that DepT, CycT, IrrT, and AnxT are positively correlated with DepS while only AnxT can independently predict the severity of DepS in patients with MDD [20]. This finding is consistent with those of previous studies conducted elsewhere [21,22]. ATs (DepT, CycT, IrrT, and AnxT) also mediate the impact of childhood abuse on adulthood DepS in patients with MDD [20]. Therefore, these ATs are considered a mediator of DepS in patients with MDD. However, the role of ATs in the psychopathology of MDD remains unknown.

From the perspective of human behavior, there are two distinct systems that provide the basis for human action: behavioral activation system (BAS) and behavioral inhibition system (BIS) [23]. The BIS and BAS are relevant to the motivation of avoiding aversive outcomes and the pursuance of goal-oriented outcomes, respectively [23]. Notably, individuals with MDD exhibit overactive BIS and deficient BAS functioning [24]. ATs and BIS/BAS are complementary but distinct entities in individuals with mood disorders [25]. Nevertheless, the relationship between ATs and BIS/BAS in individuals with MDD is not well understood.

Previously, the effects of the interactions among BIS, BAS, temperament, and other factors on DepS have been investigated. A nonclinical sample was employed to examine the effects of the interactions between ATs and stress [26], the quality of childhood parental bonding [27], and functional disability [28] on DepS. Furthermore, the effects of the interaction between BIS/BAS and stress on DepS were investigated, and a negative effect of the interaction between BAS and negative life events on DepS was reported [29]. ATs exhibit stronger effects on mood disorders than BIS/BAS on DepS [25]. However, the effects of the interaction between ATs and BIS/BAS on DepS in individuals with MDD have not been thoroughly investigated. Therefore, this study aimed to investigate the interaction effects using hierarchical multiple regression analysis.

## 2. Materials and Methods

### 2.1. Study Design and Setting

Individuals were invited to participate in this cross-sectional study using a convenience sampling method at Hokkaido University Hospital in Japan from July to December 2011. This study examined the dataset of Hokkaido University in Sapporo City. Patients are referred to Hokkaido University Hospital from across Japan as it is a tertiary medical institution. Several questionnaires were distributed among the dataset of the study to evaluate ATs, DepS, and BIS/BAS and to collect information regarding the demographic characteristics of the participants, including sex, age, education, marital and employment statuses, and familial psychiatric history. The completed questionnaires were returned anonymously via mail to our study group to ensure confidentiality. This study was approved by the Ethics Committee of Hokkaido University Hospital (Approval Number: 010-0041) and followed the Declaration of Helsinki. After thoroughly explaining the research, all participants provided written informed consent. Both outpatients and inpatients were included. The inclusion criteria were as follows: (1) diagnosed with MDD using the Diagnostic and Statistical Manual of Mental Disorders, Fourth Edition, Text Revision (DSM-IV-TR); (2) adults aged ≥20 years; (3) being able to perform the assessments; and (4) being able to consent to participate in the study. The exclusion criteria for this study were as follows: (1) being unable to perform the assessments owing to a serious mental condition; (2) having an organic psychiatric illness; (3) having a substance-use disorder; and (4) having an axis II disorder, diagnosed using DSM-IV-TR. This research is part of more extensive studies wherein some scales were used [26,30].

### 2.2. Demographic Data

The demographic information of the participants, including their age, sex, marital status, educational level, employment status, familial psychiatric history, and type of MDD, were recorded.

## 3. Assessments

DepS, ATs, and BIS/BAS were evaluated using the PHQ-9, TEMPS-A, and BIS/BAS scales, respectively.

### 3.1. Depressive Symptoms

The PHQ-9 is a valid and reliable tool for assessing depression severity [31] and is widely used in mental health assessment to screen for depression [32]. The Japanese version has been validated [33] and used to assess depression severity in patients with MDD [34]. The PHQ-9 used here comprised nine items scored on a 4-point scale from 0 to 3, and the optimal cutoff point of ≥10 had a sensitivity of 92.5% and specificity of 77.0% in the Japanese version [34], similar to the original version (sensitivity of 88% and specificity of 88% for MDD) [31]. In this study, the PHQ-9 scores ranged from 0 to 27, with lower scores indicating mild DepS [31,34].

#### 3.1.1. Affective Temperaments

The TEMPS-A is a yes-or-no questionnaire comprising 110 items designed to assess temperamental variation [15]. Cronbach’s *α* coefficients for DepT, CycT, HypT, IrrT, and AnxT were 0.69, 0.84, 0.79, 0.83, and 0.87, respectively, for the Japanese 110-item version of TEMPS-A [35]. Cronbach’s *α* for a 39-item version of TEMPS-A with 12 CycT, eight DepT, eight IrrT, eight HypT, and three AnxT subscales was 0.91 (CycT), 0.81 (DepT), 0.77 (IrrT), 0.76 (HypT), and 0.67 (AnxT) [16]. The 39-item Japanese short version of the TEMPS-A was used in this study [19,36].

#### 3.1.2. Behavioral Inhibition System/Behavioral Activation System

The BIS/BAS scale is used to evaluate the sensitivity of behavioral inhibition/activation tendencies [23]. It comprises 20 items, each rated on a 4-point Likert scale from 1 (*strongly disagree*) to 4 (*strongly agree*). The BIS scale comprised seven items (total score range, 7–28), and the BAS scale comprised 13 items (total score range, 13–52) divided into the following subscales: representing reward responsiveness (BAS–RR: five items; score range, 5–20), drive toward appetitive goals (BAS–D: four items; score range, 4–16), and fun-seeking (BAS–FS: four items; score range, 4–16). The validity and reliability of the BIS/BAS Japanese version were demonstrated, and Cronbach’s *α* was calculated as follows: 0.80 (BIS), 0.76 (BAS–D), 0.65 (BAS–FS), 0.63 (BAS–RR), and 0.81 (BAS total score) [37].

### 3.2. Statistical Analysis

**Figure 1:**
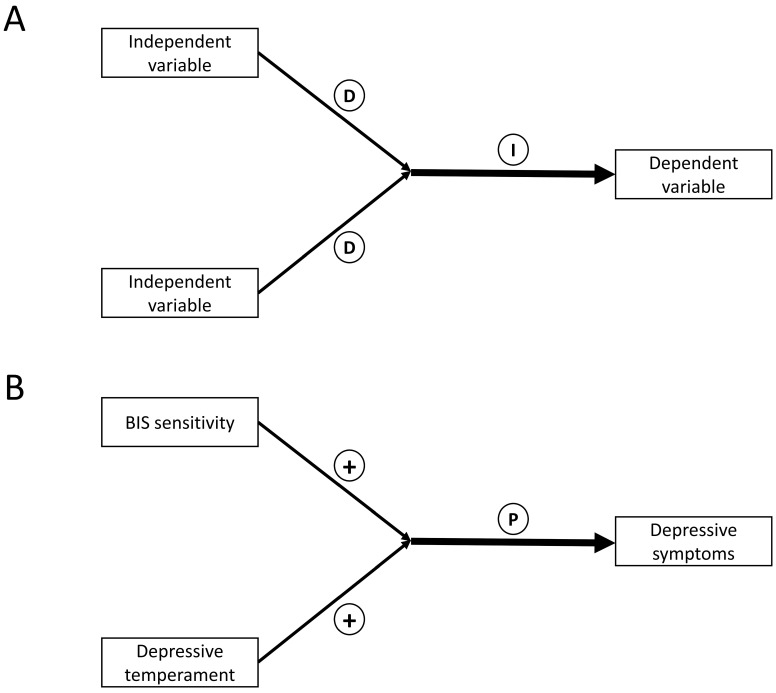
(**A**) Conceptual diagram of the interaction using moderation analysis. (**B**) Effects of the interaction between behavioral inhibition system sensitivity and depressive temperament on depressive symptoms in individuals with major depressive disorder (*N* = 90). D, direct effect; I, interaction effect; P, positive interaction effect; +, increasing the severity of depressive symptoms.

Data were stored, described, and analyzed using SPSS for Windows version 25.0 and STATA/MP 16. Pearson’s correlation analyses used Bonferroni correction to adjust probability (*p*) values to investigate the correlation among PHQ-9, TEMPS-A, and BIS/BAS scores. Multiple regression analyses (forced entry) were performed, with the PHQ-9 score as the dependent variable and the TEMPS-A and BIS/BAS scores as independent variables. Moderation analysis was employed to investigate the interaction effects (Figure 1A), and hierarchical multiple regression analysis was performed in two steps [38]. Before conducting hierarchical multiple regression analysis, the TEMPS-A and BIS/BAS scores were centered to deal with multicollinearity [39]. Factors 1 (TEMPS-A subscore) and 2 (BIS/BAS score) were examined as independent variables in the first step of hierarchical multiple regression analysis. Subsequently, an interaction term (factor 1 × factor 2) was introduced as an independent variable and analyzed alongside factors 1 and 2. The *R*^2^ change (Δ*R*^2^) was calculated from the first to the second step to evaluate the statistical significance of the interaction effect. In detail, if the Δ*R*^2^ was statistically significant, it indicated that an interaction effect was statistically significant. Two-sided *p* values were reported in this study, and *p* < 0.05 was considered statistically significant.

The moderated regression framework in the present study was as follows:

Severity of DepS (PHQ-9 score) = b0 + b1 (factor 1) + b2 (factor 2) + b3 (factor 1 × factor 2),

where

b0 is an intercept,

b1 is a coefficient related to the effect of factor 1,

b2 is a coefficient related to the effect of factor 2, and

b3 is a coefficient related to the interaction effect, which is the product of the two variables (factors 1 and 2) [26].

## 4. Results

### 4.1. Sample Characteristics

The primary findings are presented in Table 1. The mean (*SD*, standard deviation) age was 46.19 (10.73), and the average number of years of education was 13.91 (2.43). Of the 90 participants, 60 (66.67%) were male, 56 (62.22%) were married, 61 (67.78%) were currently employed, and 34 (37.78%) had a familial psychiatric history. In terms of the MDD type, 40 (44.44%) were diagnosed with a single episode of MDD and 50 (55.56%) with recurrent episodes of MDD at the time of assessment.

The clinical assessment scores are also mentioned in Table 1. The mean (*SD*) PHQ-9 score was 9.77 (6.84), close to the cutoff point [34]. The mean (*SD*) scores for TEMPS-A DepT was 1.49 (0.21), for TEMPS-A CycT was 1.29 (0.23), for TEMPS-A IrrT was 1.18 (0.16), and for TEMPS-A AnxT was 1.39 (0.25), which were higher than the average values of the same scores of individuals without MDD. The mean (*SD*) TEMPS-A HypT score was 1.18 (0.17), which was nearly identical to that of individuals without MDD [36]. The mean (*SD*) scores for BIS was 20.77 (5.13), for BAS–D was 9.97 (2.95), and for BAS–RR was 14.10 (3.13), which were nearly identical to those of individuals without MDD. The mean (*SD*) scores for total BAS was 32.62 (7.45) and for BAS–FS was 8.49 (2.51), which were lower than the average values of the scores of individuals without MDD [37].

### 4.2. Correlations between Measures

The results of Pearson’s correlation analyses are demonstrated in Table 2. The correlation analyses revealed that higher PHQ-9 scores correlated with higher DepT (*r* = 0.47, *p* < 0.001), CycT (*r* = 0.35, *p* = 0.047), IrrT (*r* = 0.45, *p* < 0.001), AnxT (*r* = 0.50, *p* < 0.001), and BIS (*r* = 0.43, *p* = 0.001) scores. The PHQ-9 scores did not significantly correlate with HypT, total BAS, BAS–D, BAS × RR, and BAS–FS scores.

### 4.3. Moderation Analysis (N = 90)

The results of moderation analysis are demonstrated in Table 3 and Figure 1B. A significant positive interaction (*β* = 0.199, *p* = 0.033) between DepT scores (*β* = 0.319, *p* = 0.004) and BIS scores (*β* = 0.300, *p* = 0.007) was observed, which had an effect on PHQ-9 scores (Δ*R*^2^ = 0.038, *p* = 0.033). However, no other combinations of BIS/BAS scores and TEMPS-A subscores exhibited a significant effect on PHQ-9 scores.

The variable represents the standardized regression coefficient (standardized *β*), except Δ*R*^2^ and adjusted *R*^2^. Table 3 shows the statistical variables from the second step but not from the first.

## 5. Discussion

We believe that the effect of the interaction between BIS sensitivity and DepT increases the severity of DepS in individuals with MDD. Notably, no significant interaction was observed between BIS sensitivity and other ATs and between BAS sensitivity and any ATs. This is the first study to report the effect of the interaction between BIS and DepT on DepS in Japanese patients with MDD. Theoretically, on the time axis, trait markers exist earlier than state markers. Therefore, ATs and BIS/BAS precede DepS. Based on this theory, we discussed how the interaction between ATs and BIS/BAS affected DepS.

From the perspective of AT, the findings of this study suggests that DepT interacts with BIS sensitivity, thereby affecting DepS in the individuals with MDD. DepT has also been related to having a history of childhood abuse and working disability, which increases the severity of DepS in adults [26,28]. DepT varies between individuals with and without MDD, with it being higher in the former than in the latter [18]. Therefore, DepT may interact with childhood abuse and working disability, thereby affecting DepS in individuals with MDD, and the interaction effects may be stronger than in individuals without MDD. In the future, we want to further investigate these interaction effects in individuals with MDD.

Regarding BIS sensitivity, our results indicate that BIS interacts with DepT, thereby affecting DepS in individuals with MDD. Although a previous study found that individuals with MDD exhibited higher BIS sensitivity than those without MDD [24], the BIS sensitivity of our participants (BIS total score = 20.77 ± 5.13) was similar to that of individuals without MDD (BIS total score = 21.38 ± 4.17) [37]. Of the participants, 55.56% were diagnosed with recurrent MDD, which may have influenced the BIS sensitivity results. In general, recurring psychopathology or depression can indicate a more severe form of MDD. Furthermore, the high number of recurrences may suggest bipolarity [40]. Hence, the possibility of bipolarity could prevent increased BIS sensitivity [41,42]. Despite this limitation, our findings suggest that BIS enhances the depressogenic effect of DepT, even in the group exhibiting moderate BIS sensitivity in MDD. According to a previous study, higher BIS sensitivity prevents the improvement of symptoms in individuals with MDD [43]. Furthermore, the effects of the interaction between BIS and DepT may prevent MDD improvement by increasing the depressogenic effect of DepT. In the future, we want to conduct a longitudinal study to investigate these causal relationships.

The effects of the interaction between BIS sensitivity and DepT can be associated with the treatment responsiveness of individuals with MDD. Personality characteristics, such as low reward dependence or cooperativeness, have been considered risk factors for treatment-resistant MDD [44]. However, the influence of the interaction between ATs and BIS/BAS on the response rates and/or remission rates in MDD has not been investigated. Recently, the use of precision psychotherapy to achieve personalized psychiatry has been receiving attention [45]. A previous study suggests that the sensitivity change in BIS/BAS is associated with responsiveness to antidepressants and remission rates in individuals with MDD [46].

Furthermore, psychological intervention may be effective for depression; however, no significant changes in BIS/BAS sensitivity were observed [47]. Therefore, the BIS/BAS sensitivity change may be more susceptible to pharmaceutical rather than psychotherapeutic interventions. These data suggest that individuals with MDD and high BIS sensitivity and DepT may be resistant to both antidepressant treatment and psychotherapy. In future studies, we would like to investigate whether the change in BIS/BAS sensitivity in response to pharmacological and psychological interventions could be differentiated using ATs in individuals with MDD. Additionally, a future longitudinal study is warranted to investigate the prognosis of individuals with Midland both high BIS sensitivity and DepT.

The current research has several limitations:

1. Owing to the cross-sectional design of the study, we could not determine the causal relationships among the variables.
2. Because our sample included twice as many men as women and was recruited in a tertiary care setting, the generalizability of our findings to all individuals with MDD may be restricted.
3. All the study participants were adults, thereby preventing generalization to individuals under the age of 20 years.
4. Memory bias could have influenced the results of the self-reported assessments used in this study. Although most questions in these assessments were regarding the last few weeks or days, negative autobiographical memories caused by depression may have influenced the results of the questionnaires used in this study.
5. The effects of medication on the results were not considered.
6. The present study was conducted in Japan. Hence, generalizability to other countries may be limited.
7. This study lacked information regarding genes associated with MDD [48].
8. A previous study suggested that affective temperament and BIS/BAS are complementary but distinct entities [25]. Neuroticism may exhibit confounding effects on the relationship between BIS/BAS–AT–depression and between BIS–AT–depression [25]. However, we did not assess neuroticism in the present study. Hence, the regression models could not be extended, which could constitute a limitation of this study.

Despite these limitations, this is the first study regarding the effects of the interaction between BIS/BAS and ATs on DepS in individuals with MDD.

## 6. Conclusions

This study suggests that the effects of the interaction between BIS sensitivity and DepT worsen DepS in individuals with MDD. In a clinical setting, such interactions may be useful in addressing DepS associated with both BIS sensitivity and DepT. Our study presents important insights into the psychopathology of individuals with MDD.

## Figures and Tables

**Table 1:** Basic findings (*N* = 90).

Demographic Characteristics	Mean (*SD*) or *N* (%)
Age, mean	46.19 (10.73)
Sex (male/female), *n*	60/30 (66.67/33.33)
Married, *n*	56 (62.22)
Years of education, mean	13.91 (2.43)
Currently employed, *n*	61 (67.78)
Familial psychiatric history, *n*	34 (37.78)
Type (single episode/recurrent), *n*	40/50 (44.44/55.56)
Scale	Mean (*SD*)
PHQ-9	9.77 (6.84)
TEMPS-A	
DepT	1.49 (0.21)
CycT	1.29 (0.23)
HypT	1.18 (0.17)
IrrT	1.18 (0.16)
AnxT	1.39 (0.25)
BIS	20.77 (5.13)
BAS total	32.62 (7.45)
BAS–D	9.97 (2.95)
BAS–RR	14.10 (3.13)
BAS–FS	8.49 (2.51)

Note. BAS, behavioral activation system; BIS, behavioral inhibition system; D, drive; FS, fun-seeking; MDD, major depressive disorder; PHQ-9, Patient Health Questionnaire-9; RR, reward responsiveness; RUD, recurrent unipolar depression; *SD*, standard deviation; TEMPS-A, Temperament Evaluation of Memphis, Pisa, Paris, and San Diego Auto-questionnaire (depressive [DepT], cyclothymic [CycT], hyperthymic [HypT], irritable [IrrT], and anxious [AnxT] temperaments).

**Table 2:** Pearson’s correlation analysis (*N* = 90).

	1	2	3	4	5	6	7	8	9	10
1. PHQ-9	—									
2. DepT	0.47 ***	—								
3. CycT	0.35 *	0.41 **	—							
4. HypT	−0.08	−0.09	0.06	—						
5. IrrT	0.45 ***	0.51 ***	0.60 ***	−0.06	—					
6. AnxT	0.50 ***	0.61 ***	0.60 ***	−0.15	0.68 ***	—				
7. BIS	0.43 **	0.54 ***	0.40 **	−0.23	0.41 **	0.57 ***	—			
8. BAS total	0.10	0.12	0.20	0.44 **	0.15	0.10	0.20	—		
9. BAS–D	0.05	0.08	0.13	0.39 **	0.15	0.07	0.15	0.87 ***	—	
10. BAS–RR	0.09	0.19	0.18	0.35 *	0.06	0.12	0.30	0.88 ***	0.64 ***	—
11. BAS–FS	0.12	−0.01	0.22	0.42 **	0.16	0.03	−0.01	0.83 ***	0.58 ***	0.60 ***

* *p* < 0.05, ** *p* < 0.01, *** *p* < 0.001.

**Table 3:** Moderation analysis (*N* = 90).

	Dependent Factor: PHQ-9 Score				
	Affective Temperaments				
Independent variables	DepT	CycT	HypT	IrrT	AnxT
TEMPS-A score	0.319 **	0.197	−0.005	0.315 **	0.370 **
BIS	0.300 **	0.358 **	0.439 ***	0.308 **	0.254 *
Interaction	0.199 *	0.014	−0.075	0.044	0.134
Δ*R*^2^	0.038 *	0.000	0.005	0.002	0.017
Adjusted *R*^2^	0.280	0.197	0.166	0.254	0.276
	DepT	CycT	HypT	IrrT	AnxT
TEMPS-A score	0.484 ***	0.341 **	−0.174	0.456 ***	0.498 ***
BAS total	0.055	0.025	0.166	0.024	0.047
Interaction	0.126	−0.008	0.046	0.081	0.022
Δ*R*^2^	0.015	0.000	0.002	0.006	0.000
Adjusted *R*^2^	0.209	0.089	−0.006	0.180	0.226
	DepT	CycT	HypT	IrrT	AnxT
TEMPS-A score	0.470 ***	0.344 **	−0.146	0.457 ***	0.502 ***
BAS–D	0.006	0.003	0.096	−0.029	0.011
Interaction	0.107	0.009	0.071	0.077	0.044
Δ*R*^2^	0.011	0.000	0.004	0.006	0.002
Adjusted *R*^2^	0.204	0.089	−0.018	0.179	0.226
	DepT	CycT	HypT	IrrT	AnxT
TEMPS-A score	0.493 ***	0.341 **	−0.109	0.462 ***	0.508 ***
BAS–RR	0.010	0.023	0.124	0.049	0.022
Interaction	0.102	0.002	−0.026	0.095	0.043
Δ*R*^2^	0.010	0.000	0.001	0.009	0.002
Adjusted *R*^2^	0.202	0.089	−0.013	0.185	0.226
	DepT	CycT	HypT	IrrT	AnxT
TEMPS-A score	0.493 ***	0.344 **	−0.181	0.450 ***	0.496 ***
BAS–FS	0.141	0.034	0.182	0.057	0.102
Interaction	0.143	−0.038	0.053	0.074	−0.013
Δ*R*^2^	0.019	0.001	0.002	0.005	0.000
Adjusted *R*^2^	0.227	0.092	0.002	0.180	0.235

* *p* < 0.05, ** *p* < 0.01, *** *p* < 0.001.

## Data Availability

The datasets generated during and/or analyzed during the current study are available upon reasonable request from the corresponding author.
